# Discovery of new fluorescent thiazole–pyrazoline derivatives as autophagy inducers by inhibiting mTOR activity in A549 human lung cancer cells

**DOI:** 10.1038/s41419-020-02746-w

**Published:** 2020-07-20

**Authors:** ZhaoMin Lin, ZhaoYang Wang, XueWen Zhou, Ming Zhang, DongFang Gao, Lu Zhang, Peng Wang, Yuan Chen, YuXing Lin, BaoXiang Zhao, JunYing Miao, Feng Kong

**Affiliations:** 1 Institute of Medical Science, The Second Hospital of Shandong University, Jinan, 250033 PR China; 2 Shandong Provincial Key Laboratory of Animal Cells and Developmental Biology, School of Life Science, Shandong University, Jinan, 250100 PR China; 3 Institute of Organic Chemistry, School of Chemistry and Chemical Engineering, Shandong University, Jinan, 250100 PR China; 4 Department of Central Laboratory, Shandong Provincial Hospital affiliated to Shandong University, Jinan, 250021 PR China

**Keywords:** Autophagy, Target identification

## Abstract

A series of fluorescent thiazole–pyrazoline derivatives was synthesized and their structures were characterized by ^1^H NMR, ^13^C NMR, and HRMS. Biological evaluation demonstrated that these compounds could effectively inhibit the growth of human non-small cell lung cancer (NSCLC) A549 cells in a dose- and time-dependent manner in vitro and inhibit tumor growth in vivo. The structure–activity relationship (SAR) of the compounds was analyzed. Further mechanism research revealed they could induce autophagy and cell cycle arrest while had no influence on cell necrosis. Compound **5e** inhibited the activity of mTOR via FKBP12, which could be reversed by 3BDO, an mTOR activator and autophagy inhibitor. Compound **5e** inhibited growth, promoted autophagy of A549 cells in vivo. Moreover, compound **5e** showed good selectivity with no influence on normal vascular endothelial cell growth and the normal chick embryo chorioallantoic membrane (CAM) capillary formation. Therefore, our research provides potential lead compounds for the development of new anticancer drugs against human lung cancer.

## Introduction

Cancer is still a major global health concern and a leading cause of death all over the world. It is shown that lung cancer remains the highest death rate in all cancer deaths both in developed and developing countries^1^. Over the past decades, much attention has been paid to the discovery of effective method to overcome cancer thoroughly. Despite more and more anticancer therapies were developed, chemotherapy is still one of the most common cancer therapies to prolong the lifespan of cancer patients^2,3^. However, due to side effect and drug resistance, it is an urgent issue to develop novel, selective anticancer agents.

Nevertheless, studying the distribution and targets of anticancer compounds in living cells poses a great challenge for researchers and great help to improve the activity and selectivity. Fluorescigenic small molecules provide a huge boost for determining their location and targets in living cells. Fluorescent compounds have been used as powerful detection tools in cell biology. Currently, due to the nature of high quantum yield and readily synthetic process, some pyrazoline derivatives have been synthesized and used in fluorescence probes, for orientation^4^, detecting cation^5–8^, hydrazine^9,10^, thiols^11–13^, and DNA^14^. Moreover, their biological roles have been studied in insecticidal function^15–17^, human monoamine oxidase activity inhibition^18,19^, anti-inflammation^20–22^, antimicrobial^23,24^, analgesia^25^. In addition, pyrazoline derivatives could inhibit the proliferation of cancer cells with satisfactory activity^26,27^. However, the anticancer mechanism was little delineated.

Autophagy, an important process in eukaryotes through which useless organelles were delivered to lysosomes for degradation and reuse, plays double-edged roles in tumor initiation and progression depending on different cell types and specific stages of tumor progression^28,29^. On the one hand, autophagy deficiency has a positive effect on malignant transformation, indicating autophagy as a tumor suppressor mechanism^30,31^. On the other hand, excessive autophagy could contribute to cell death in certain cancer cell types which maintained the cellular functions by triggering autophagy^32,33^. Considering the dual nature of autophagy in tumorigenesis and progression, more modulators of autophagy may provide a powerful tool for cancer therapy.

Mechanistic target of rapamycin (mTOR [serine/threonine kinase]/FK506-binding protein 12-rapamycin associated protein 1), regulates the maintenance of cell homeostasis, including cell growth, autophagy, and cytoskeletal organization^34,35^. The dysregulated activity of mTOR involved in several human disorders, including cancers, such as lung cancer, breast cancer, and others^36^. Due to the key role of proliferation in numerous malignant cell types, there were many potential applications in the therapy of various solid tumors and hematological malignancies by targeting the mTOR pathway^37,38^. However, the expectations of more effective and less toxic treatment with mTOR inhibitors have not realized.

In a continuation of an ongoing program aiming at finding novel fluorescent small molecules with anticancer activity^39–41^, a series of thiazole–pyrazoline derivatives were synthesized and their properties in A549 cells were evaluated. In this work, deep insights into the antineoplastic activity and mechanism of pyrazoline derivatives were gained to provide a basis for the rational and targetable design of fluorescent anticancer drug for clinical application.

## Materials and methods

### Reagents and apparatus

All reagents were of analytical grade or chemically pure. Thin-layer chromatography (TLC) was performed on silica gel 60 F_254_ plates (Merck KGaA) and column chromatography was conducted over silica gel (mesh 200–300). ^1^H NMR spectra were recorded on a Bruker Avance 400 (400 MHz) spectrometer or Bruker Avance 300 (300 MHz) spectrometer, using DMSO-d6 as solvent and tetramethylsilane as an internal standard. Melting points were determined on an XD-4 digital micro melting point apparatus. IR spectra were recorded with an IR spectrophotometer Avtar 370 FT-IR (Termo Nicolet). MS spectra were recorded on a Trace DSQ mass spectrograph. Unless otherwise stated, all reagents were purchased from J&K, Sinopharm Chemical Reagent Co. and Kermel and used without further purification. Twice-distilled water was used throughout all experiments. Rapamycin was from Calbiochem (Darmstadt, Germany). Chloroquine (CQ) and Bafilomycin-A1 (Baf-A1) were purchased from Sigma-Aldrich (St. Louis, MO, USA).

### Preparation of chalcone compounds (**3**)

In a flask, compound **1** (10 mmol) was dissolved in ethanol (10 ml), and sodium hydroxide solution in water (8 ml, 2.5 M) was added. Then compound **2** dissolved in ethanol (10 ml) was added to the above mixture. After reaction completed (by TLC monitoring), yellow precipitate was filtered, then the solid was washed with water to make pH = 7. The yellow product was dried by infrared lamp to give compound **3** in more than 80% yield.

### Preparation of 3,5-diaryl-4,5-dihydro-1H-pyrazole-1-carbothioamide (**4**)

Thiosemicarbazide (6 mmol), sodium hydroxide (8 mmol), compound **3** and ethanol (30 ml) were added into round-bottom flask. The reaction mixture was refluxed for 3–8 h, it was monitored by TLC until completion. The mixture was cooled to room temperature. Precipitate was filtered, and washed three times with water and ethanol. After dried with infrared lamp, corresponding product **4** was obtained.

### Preparation of compound **5**

Compound **4** (1 mmol) was added into ethanol (25 ml), then 2-bromo-1-(pyridin-4-yl)ethanone (1 mmol) was added into the mixture. The reaction mixture was refluxed for 4–9 h (monitored by TLC until completion). The mixture was cooled to room temperature and filtered. The solid was washed with ethanol two times. The solid was dissolved in dichloromethane. The mixture was washed with saturated NaHCO_3_, followed by wash with saturated brine. Organic phase was dried over magnesium sulfate. After desiccant was removed by suction filter, organic phase was concentrated under reduced pressure to give the target products **5**. The spectroscopy data of compounds **5a**–**5j** are loaded in the Supplementary file.

### Antibodies

Antibody for Light chain 3 beta (LC3B) (2775 S), EIF4EBP1 (9452), p-EIF4EBP1 (9459), RPS6KB1 (9202), p-RPS6KB1 (9205), and p-mTOR (2971) were purchased from CST. Antibody for β-actin (sc-47778), mTOR (sc-8319), FKBP12, and horseradish peroxidase-conjugated secondary antibodies were bought from Santa Cruz. Secondary antibodies for immunofluorescence were donkey anti-rabbit IgG Alexa Fluor-488 (A10040), which was purchased from life technology.

### Cell culture

All the cells utilized in the experiment were purchased from the Cell Culture Bank of the Chinese Academy of Sciences (http://www.cellbank.org.cn/). Human lung cancer cell line A549 and H460, human liver carcinoma cell line HepG-2, human hormone-independent prostate carcinoma cell line PC3, human kidney clear cell adenocarcinoma cell line 786-O, human breast carcinoma cell line 4T1, and human renal tubular epithelial cell HK-2 were cultured in RPMI-1640 medium with 10% (v/v) bovine calf serum and 80 U/ml penicillin/streptomycin. Human glioblastoma cells U87 and human embryonic kidney cell 293T were grown in DMEM medium (Gibco, USA) with 10% FBS, penicillin (50 U/ml), and streptomycin (50 ug/mL) (Invitrogen, 10378-016). Human umbilical vein endothelial cells (HUVEC) were grown in M199 medium (Gibco, 31100-035) with 10% (v/v) bovine calf serum and 8.4 IU/mL FGF2. All cell lines were cultured in a humidified incubator with 5% CO_2_ at 37 °C.

### Cell morphology

Morphologic changes of A549 cells treated with compounds at indicated concentration for 12, 24, and 48 h were examined by inverted phase-contrast microscope (Eclipse TS-100; 21 Nikon, Tokyo).

### Cell viability assay

Cells were seeded onto 96-well plates for 24 h and then treated with 0.1% DMSO (v/v, as control), 5-fluorouracil (5-FU, as positive group) or compounds **5a**–**5j** at indicated concentrations (0.1, 1, 5, 10 μM) for 24 and 48 h. Cell viability was measured by sulforhodamine B (SRB) assay, in accordance with the previous method^42^. The intensity of light absorption was measured by using a SpectraMAX190 microplate spectrophotometer (GMI Co, USA) at the wavelength of 540 nm. In some experiments, A549 cells were transfected with siRNAs and/or treated with **5e**. A549 cells were exposed to autophagy inhibitors CQ (20 mM) and Baf-A1 (50 nM) for 2 h before treatment with **5e**.

### Lactate dehydrogenase (LDH) assay

Cell culture medium was gathered after 24-h treatment with compounds **5a**–**5j** (10 μM) or 0.1% DMSO (as control). LDH assay was conducted by using a LDH kit (Nanjing Jiancheng Co, China), according to the manufacturer’s description.

### Co-localization imaging of cells

A549 cells were incubated with **5e** (1 μM) for 1 h at 37 °C. Then, MitoTracker Deep Red (0.1 μM), Lyso Sensor Green (0.3 μM), and ER Tracker Red (0.3 μM) were added and incubated for another 0.5 h and the confocal fluorescent images were captured.

### Western blot analysis

Total proteins were obtained from A549 by using IP lysis buffer (Shanghai beyotime Co., China) after different treatment. Cells were washed twice with ice-cold phosphate-buffered saline (PBS), then lysed in protein lysis buffer (Shanghai beyotime Co., China). The protein concentration of the cells was measured by the Bradford method. Following separation by SDS-PAGE and transferring to PVDF membrane (Millipore, USA), proteins were incubated with primary antibodies, then incubated with horseradish peroxidase-linked secondary antibodies, and finally probed by using an enhanced chemiluminesence detection kit (Thermo). Actin β was used as a loading control. The relative quantity of proteins was analyzed by Image J software and normalized to loading controls.

### RNA interference

A549 cells were transiently transfected with siRNA duplex oligonucleotides targeting LC3B (GenePharmcon, Shanghai, China) using Lipofectamine 2000 (Invitrogen, 11668-019). After 24-h transfection, cell lysates were subjected for the western blot assay and cells were treated with **5e** or vehicle for an additional 24 h. Cell viability was determined as described above. FKBP12 siRNA (sc-35678) and scramble RNA (sc-37007) were obtained from Santa Cruz Biotechnology. A549 cells at 50–60% confluence were transfected with 60-nM siRNA against FKBP25, FKBP12, and scramble siRNA with Lipofectamine 2000 according to the manufacturer’s instructions. Then cells were harvested and analyzed by western blot.

### Flow cytometric analysis of cell cycle distribution

Following treated with compounds **5a**, **5d**, **5e**, **5g** and **5h** (10 μM) for 48 h, A549 cells were harvested and fixed with 70% ice-cold ethanol, then stained with 50 mg/ml propidium iodide containing 10 mg/ml RNase A at 4 °C for 10 min. The stained cells were analyzed by using a flow cytometer (ImageStream^X^ MarkII, Amnis, USA). The cell cycle distribution was analyzed by IDEAS software (Amnis, USA).

### Immunofluorescence assay

Treated cells were fixed in 4% paraformaldehyde (w/v) for 30 min at room temperature and then incubated with normal donkey serum (1:30) for 30 min and primary antibodies (1:100) overnight at 4 °C. Cells were washed with PBS times, and then three incubated with secondary antibodies (1:200) for 1 h at 37 °C. Fluorescence was detected by laser scanning confocal microscopy Zeiss LSM700 (Germany). Frozen sections of tumors formed on the chick embryo chorioallantoic membrane (CAM) were fixed with cold acetone for 10 min and blocked with 10% normal donkey serum (Solarbio, SL050) for 30 min at room temperature. Then frozen sections of tumors were incubated with primary antibody (1:100; LC3B, Rabbit polyclonal antibody, Santa Cruz Biotechnology) at 4 °C overnight and then corresponding secondary antibody (1:200) at 37 °C for 1 h. Frozen sections of tumors were washed three times with 0.1-M PBST. DAPI (1:200) was added to stain cell nucleus for 10 min and then the sections were washed three times with PBS. Fluorescence was detected by confocal fluorescence microscopy Zeiss LSM700 (Germany).

### Chick embryo CAM assay

Fertile chicken eggs (7–9 days old) were used to conduct the CAM assay. An amount of (1–10) × 10^6^ A549 cells suspended in 20-μL RPMI-1640 was engraftment on the CAM. Next, the egg shell was sealed with gas-permeable tape to avoid bacterial infection for another two days. Then, the eggs were treated with PBS (negative control, qod × 3), the compound **5e** (25 and 50 μM, qod × 3) or 5-Fu (50-μM positive group, qod × 3). After fixed by 4% paraformaldehyde for 30 min, CAMs were separated from the eggs and photographed by a stereomicroscope (Japan).

### Angiogenesis assay of CAM in vivo

Fertilized chicken eggs were incubated with 55% relative humidity at 37 °C. On embryonic day 7 or 8, **5**e (25 and 50 μM) soaked in the gelatin sponge was applied to the CAM and DMSO as the vehicle control for 48 h. Then, repeated the above operation for three times. At the end of the incubation, after fixed by 4% paraformaldehyde for 30 min, the CAM zones around the gelatin sponge were photographed and analyzed by using the Image-Pro Plus.

### Statistical analyses

Data were presented as means ± SE and analyzed by SPSS software. Pictures were processed with Photoshop software. Mean values were derived from at least three independent experiments. Differences at *p* < 0.05 were considered statistically significant.

## Results

### Chemistry

**Scheme 1:**
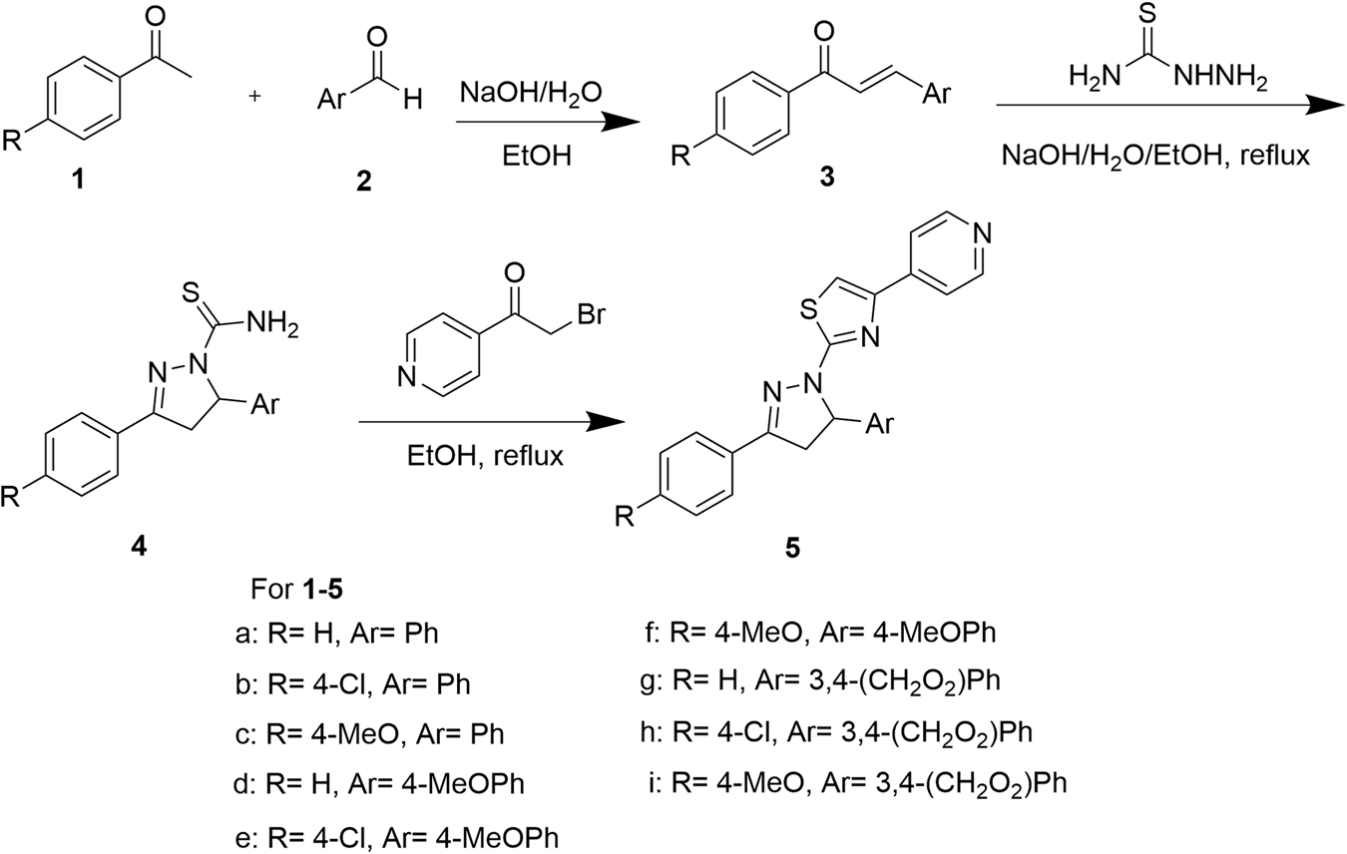
Synthesis of compounds **5a–5j**.

The synthetic route of compounds **5a**–**5j** has been accomplished as shown in Scheme 1^43^. An aryl aldehyde (**1**) reacted with an aryl methyl ketone (**2**) to give a chalcone (**3**). The chalcone (**3**) reacted with thiosemicarbazide to afford 3,5-diaryl-4,5-dihydro-1H-pyrazole-1-carbothioamide (**4**). Compound **4** reacted with 2-bromo-1-(pyridin-4-yl)ethanone to produce target products, 2-(3,5-diaryl-4,5-dihydro-1H-pyrazol-1-yl)-4-(pyridin-4-yl)thiazole (**5**).

### In vitro antiproliferative activity and structure–activity relationship (SAR) study

**Fig. 1:**
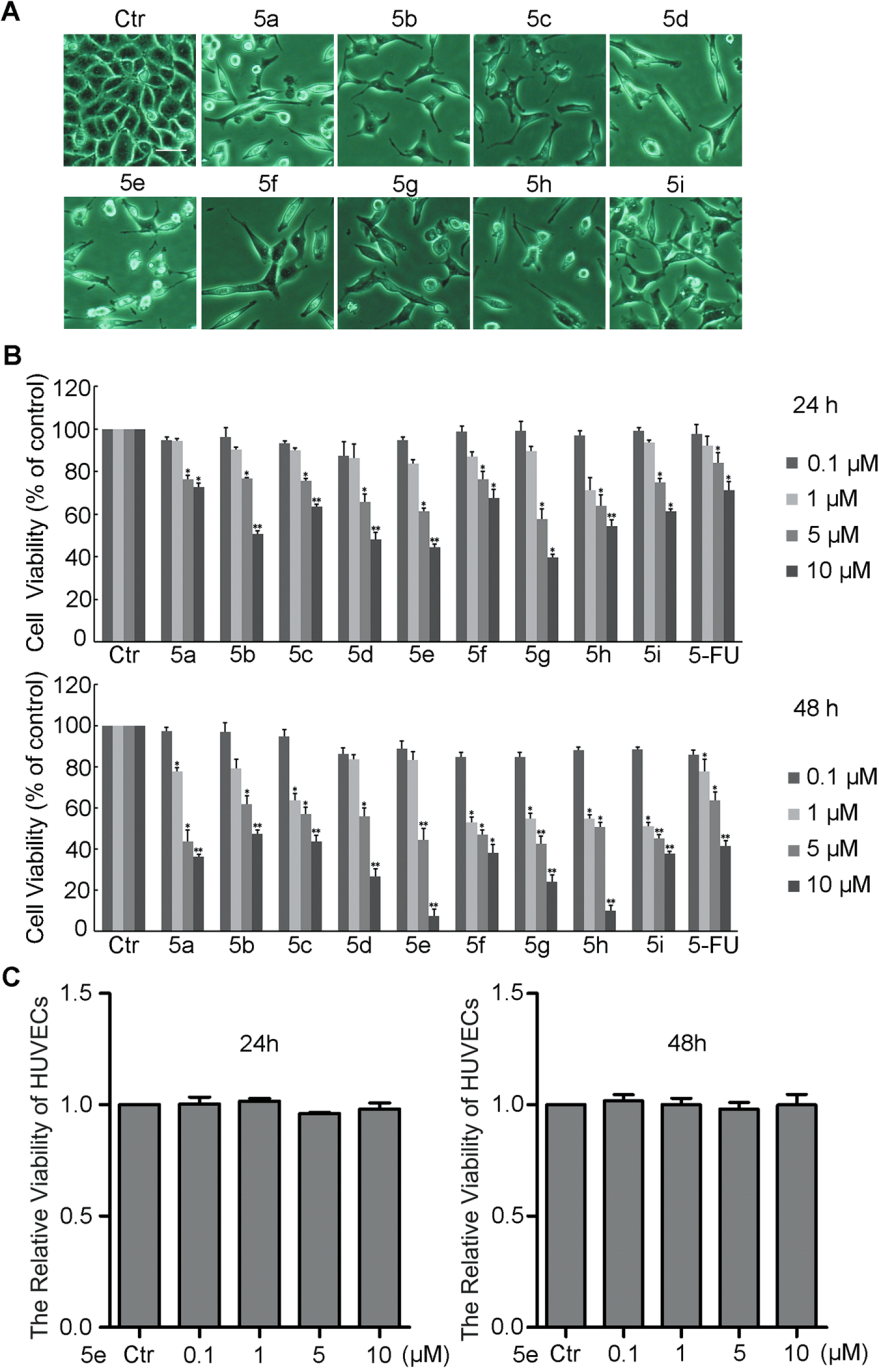
The viability of cells incubated with compounds **5a–5i**. **a** Effects of compounds **5a–5i** at 10 μM on changes in cell morphology for 48 h (100×). These images were re-used from Fig. S1. **b** Effects of compounds **5a**–**5i** on cell viability assessed by sulforhodamine B for 24 and 48 h. **c** The effect of compound **5e** on HUVECs cell viability assessed by sulforhodamine B for 24 and 48 h. Results were presented as mean ± SE; *n* = 3; *p < 0.05; **p < 0.01. Bar = 20 μm.

In order to examine the anticancer activity of compounds **5a**–**5j**, we firstly observed the morphological changes of A549 cells treated for 12, 24, or 48 h by a phase-contrast microscope. The data showed obvious morphological changes in A549 cells treated with the compounds **5a**–**5j** in dose- and time-dependent manners (Fig. S1). Compared with control group, the cell density dramatically decreased and cells were elongated or formed triangle and arborization significantly treated for 48 h (Fig. 1a, an excerpt of Fig. S1). SRB assay was conducted to investigate the effect of these compounds on cell proliferation. The data indicated that these compounds suppressed the growth of A549 cells in a dose-dependent manner after treatment with the compounds for 24 and 48 h (Fig. 1b). The IC_50_ (μM) values of the compounds were totally <10 μM (Table 1). Our finding demonstrated that all tested derivatives exhibited considerable cell growth inhibition on A549 cells. Furthermore, we explored the inhibitory effect of compound **5e** on human cancer cells lines H460, HepG-2, PC3, 786-O, 4T1, J82, and human nontumorigenic cell lines HK-2 and 293T. As shown in Table S1, compound **5e** also exhibited obvious growth inhibiting activity against other cancer cell lines, but did not inhibit the growth of normal cell lines. **5e** had no influence on the growth of HUVECs (Fig. 1c), indicating that this compound showed good selectivity.

**Table 1:** Growth inhibitory properties (IC_50_, 48 h) of compounds **5a**–**5i** and 5-FU in A549 cells. Results are mean ± SEM.

Compounds	IC_50_ (μM)	Compounds	IC_50_ (μM)
**5-FU**	9.4 ± 0.23	**5e**	2.6 ± 0.11
**5a**	4.4 ± 0.12	**5f**	2.9 ± 0.09
**5b**	8.7 ± 0.15	**5g**	1.8 ± 0.08
**5c**	6.0 ± 0.08	**5h**	1.8 ± 0.05
**5d**	4.7 ± 0.12	**5i**	2.7 ± 0.36

Further, based on the above results, the SAR of the compounds was analyzed. The antiproliferative activity of these compounds are mainly affected by aryl group in 5 position of pyrazoline moiety. When substituent Ar is benzo[d][1,3]dioxol-5-yl (compounds **5g**–**5i**), the inhibition effect for cell growth is stronger. When Ar is 4-methoxyl phenyl (**5d**–**5f**), compounds have also a higher growth inhibitory effect. However, in the case of Ar is phenyl, antitumor activity is poorer. Taken together, compounds **5g**, **5h** were the most effective compounds in suppressing A549 cell growth.

Continuous proliferation is the hallmark of cancer cells. Because regulation of the cell cycle is critical for cell growth, we investigated the effect of compounds **5** on cell cycle progression using flow cytometry. The results showed that compounds **5a**, **5d**, **5e**, **5g**, or **5h** effectively arrested the cell cycle at the G1 phase at the concentration of 10 μM for 48 h, which were in accordance with the growth inhibitory effect of these compounds. Notably, treatment with **5e** enhanced the G1 population by 33.8% (Fig. S2A). Necrosis, an unwanted side effect of cancer-fighting agents, could be evaluated by the LDH assay. LDH assay was performed on cells treated with the compounds and 0.1% DMSO. Our data revealed that these compounds had no influence on the release of LDH (Fig. S2B).

### Compound 5e distributed in lysosome in A549 cells

Given these compounds containing fluorescent group, we detected the fluorescence in A549 cells by a fluorescent microscope. The data indicated that these compounds have good excellent water-solubility and membrane permeability, especially compound **5e** had good fluorescence at 0.1 μM (Fig. S3). Combining with **5e** showed good cell growth inhibition activity and high selectivity, this compound was chosen for further mechanism research.

**Fig. 2:**
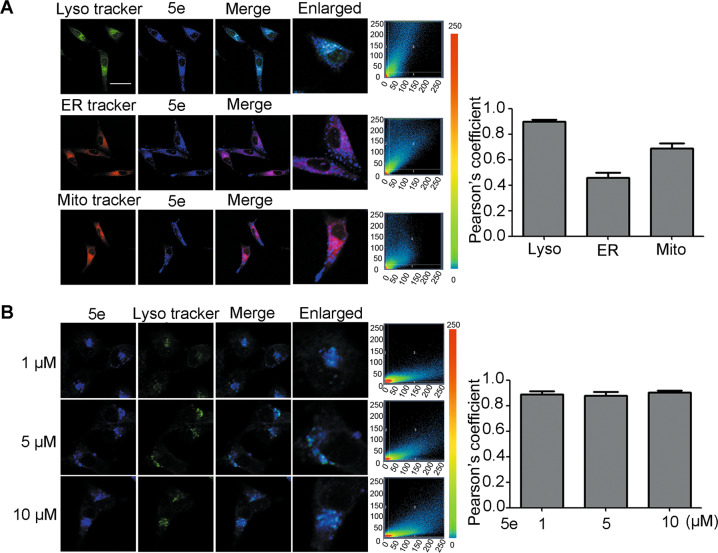
The distribution of compound **5e** in A549 cells. **a** A549 cells were treated with 10-μM compound **5e**, followed by MitoTracker Deep Red (0.1 μM, 0.5 h), Lyso Sensor Green (0.3 μM, 0.5 h), or ER Tracker Red (0.3 μM, 0.5 h). The average Pearson’s coefficient was shown in bar chart. Compound **5e**: *λ*_ex_ = 405 nm, *λ*_em_ = 405–490 nm. MitoTracker Deep Red: *λ*_ex_ = 635 nm, *λ*_em_ = 635–700 nm. Lyso Sensor Green: *λ*_ex_ = 488 nm, *λ*_em_ = 488–700 nm. ER Tracker Red: *λ*_ex_ = 555 nm, *λ*_em_ = 555–700 nm. **b** A549 cells were treated by 1 μM, 5 μM, or 10 μM compound **5e** for 24 h, then, incubated with Lyso Sensor Green (0.3 μM, 0.5 h). The average Pearson’s coefficient was shown in bar chart. Compound **5e**: *λ*_ex_ = 405 nm, *λ*_em_ = 405–490 nm. Lyso Sensor Green: *λ*_ex_ = 488 nm, *λ*_em_ = 488–700 nm (200×). *X*-axis means mean intensity of compound **5e** and *y*-axis means mean intensity of different trackers. Numbers 1 and 2 mean respective regions and 3 means colocation region. Bar = 10 μm.

First, the intracellular distribution of compound **5e** in A549 cells was explored according to good fluorescence. We used commercial lysosome probe (Lyso Sensor Green), mitochondria probe (mitochondria Deep Red), and endoplasmic reticulum probe (ER red) to co-stain A549 cells. The result showed that compound **5e** had good co-localization with lysosome (Pearson’s coefficient 0.903), but poor with mitochondria (Pearson’s coefficient 0.563) or endoplasmic reticulum (Pearson’s coefficient 0.733) (Fig. 2a), implying a preferential distribution of compound **5e** in lysosome. Furthermore, the concentration of compound **5e** had no significant effect on its cellular distribution (Fig. 2b).

### Compound **5e** induced autophagy of A549 cells

**Fig. 3:**
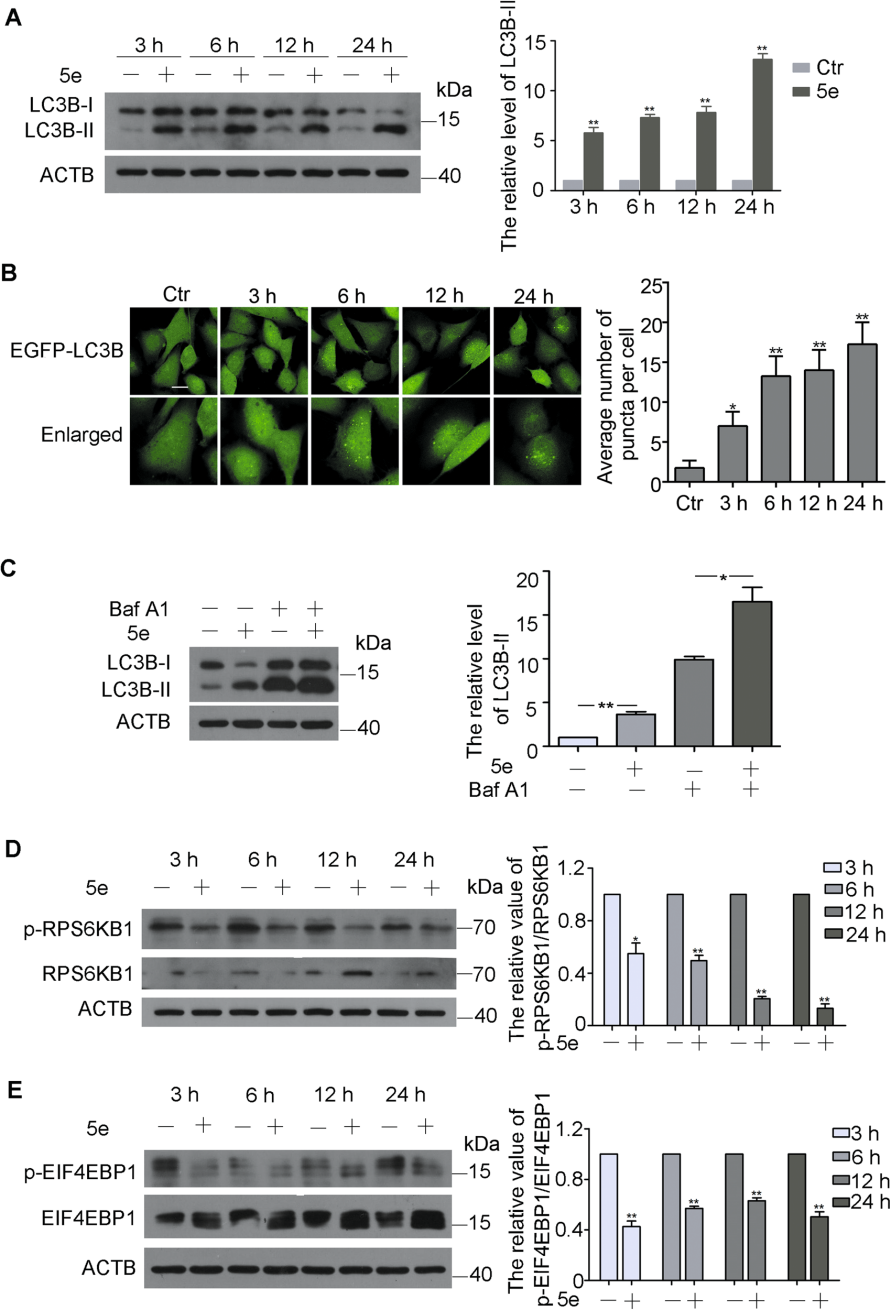
Compound **5e** induced autophagy in a mTOR-dependent manner. **a** Western blot analysis of LC3B-I and LC3B-II in A549 cells treated with compound **5e** at 10 μM for indicated times and quantification of LC3B-II levels. **b** Images of EGFP-LC3B U87 cells were treated with compound **5e** at the concentration of 10 μM for 3, 6, 12, and 24 h (200×) and quantification of EGFP-LC3B dots. Bar = 10 μm. **c** Western blot analysis of LC3B-I and LC3B-II in A549 cells treated with compound **5e** (10 μM), Baf-A1 (50 nM), or both for 12 h and quantification of LC3B-II levels. **d** Western blot analysis of RPS6KB1 and p-RPS6KB1 (S424/T421) in A549 cells treated with compound **5e** at 10 μM for indicated times and quantification. **e** Western blot analysis of EIF4EBP1 and p-EIF4EBP1 (S65/T70) in A549 cells treated with **5e** at 10 μM for indicated times and quantification. β-actin was used as a loading control. Results were presented as mean ± SE; *n* = 3; *p < 0.05; **p < 0.01.

Autophagy is a highly conserved lysosomal degradation pathway in which unnecessary byproducts and damaged organelles are engulfed into double-membrane vesicles termed autophagosomes and transported to lysosomes^44,45^. Due to compound **5e** located in lysosomes, we investigated the effect of this compound on autophagy. LC3B, an autophagy maker, was monitored by western blotting. The result showed that the levels of LC3B-II were enhanced after incubation with **5e** at 10 μM for 3, 6, 12 and 24 h, indicating compound **5e** induced autophagy in a time-dependent manner (Fig. 3a). Moreover, autophagic LC3B-II accumulation was dramatically enhanced in stably expressing EGFP-LCB3 U87 cells in a time-dependent manner (Fig. 3b). Actually, all other compounds could also induce autophagy (Fig. S4). In order to demonstrate whether compound **5e** could induce intact autophagy flux, Baf-A1, a recognized inhibitor of vacuolar H^+^-ATPase, was used to block autophagy. As shown in Fig. 3c, treatment with compound **5e** further enhanced the accumulation of LC3B-II induced by Baf-A1, implying that compound **5e** could induce complete autophagy flux. To assess the impact of autophagic flux in **5e**-induced cell death, we analyzed cell viability and cell death by pretreating A549 cells with autophagy inhibitors, chloroquine, and Baf-A1. The results showed that these two inhibitors both could increase the cell viabilities compared with cells treated with **5e** solely (Fig. S5A,B). We further confirmed the role of autophagic flux in the action of **5e** by knockdown of specific autophagy-related LC3B gene. Reduction of LC3B by siRNA significantly alleviated cytotoxic activity of **5e** (Fig. S5C,D). Taken together, our data clearly demonstrated that **5e** activated an autophagic flux and promoted autophagy-dependent cell death. Compound **5e** had no influence on the growth of HUVECs and could not induce autophagy in HUVECs (Fig. S6).

mTOR is a crucial molecular during the process of autophagy. To understand whether compound **5e** can suppress the activity of mTOR, we examined the influence of compound **5e** on the phosphorylation of RPS6KB1 (ribosomal protein S6 kinase, 70 kDa, polypeptide 1) and EIF4EBP1 (eukaryotic translation initiation factor 4E-binding protein 1), two essential substrates of mTOR^36,37^. Obviously, the levels of phosphorylation of RPS6KB1 and EIF4EBP1 were significantly decreased after treatment with compound **5e** for 3, 6, 12 and 24 h (Fig. 3d, e). The levels of p-mTOR and mTOR were detected by immunofluorescence staining based on the fluorescence characteristics of compound **5e**. The data showed that **5e** could reduce the phosphorylation of mTOR (Fig. S7). Therefore, compound **5e** might induce autophagy in an mTOR-dependent manner.

### Compound **5e** targeted to FKBP12 and inhibited mTOR

**Fig. 4:**
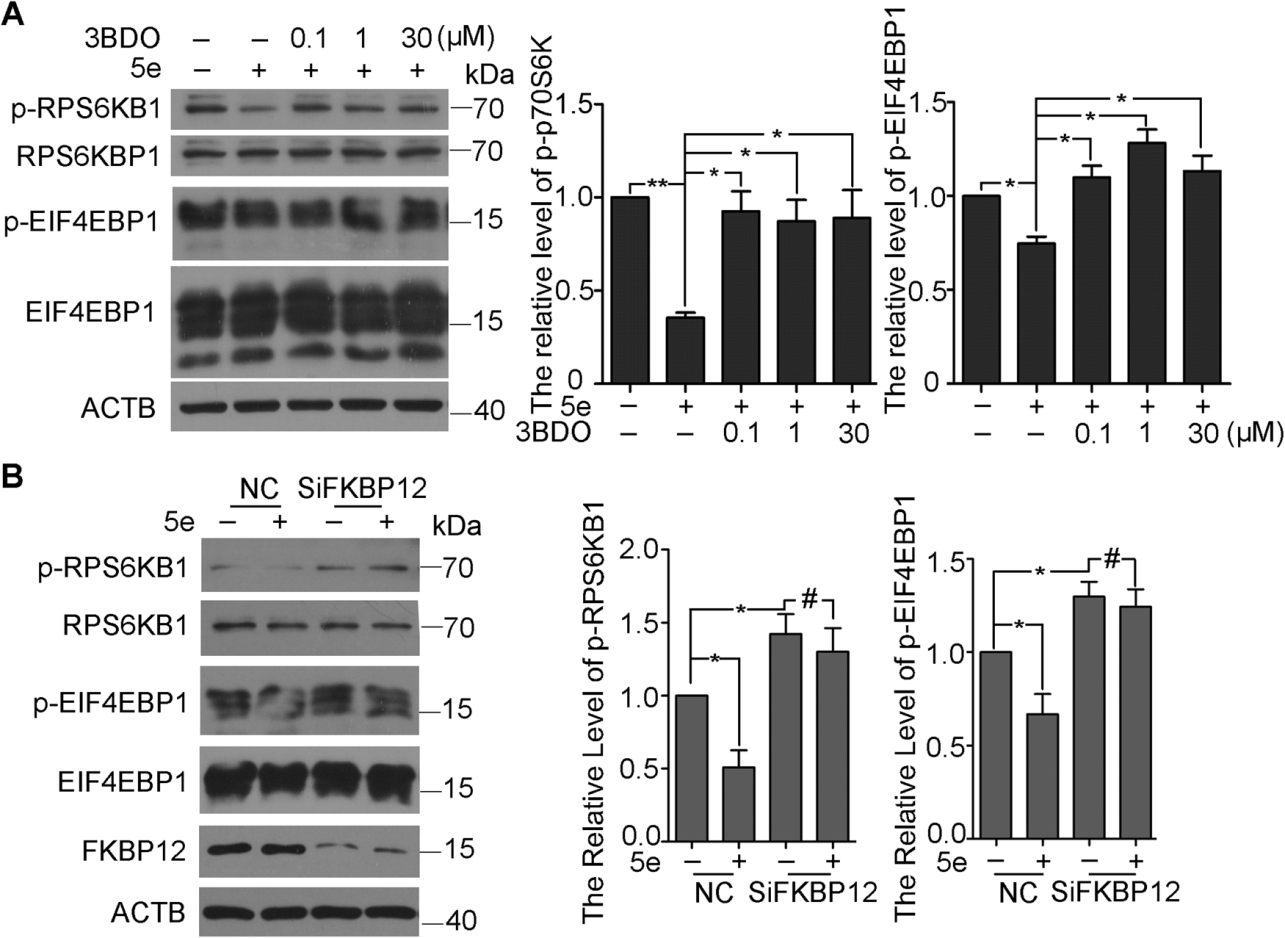
Compound **5e** inhibited mTOR activity via FKBP12. Western blot analysis of RPS6KB1, p-RPS6KB1, EIF4EBP1, and p-EIF4EBP1. **a** A549 cells were treated with compound **5e** at indicated concentrations for 6 h along with pretreatment of 3BDO or not. The pretreatment time of 3BDO was 3 h and the concentration were 0.1, 1, and 30 μM respectively. **b** A549 cells were treated with compound **5e** for 6 h in 10 μM solely or along with SiFKBP12 in 60 nM for 24 h. β-actin was used as a loading control. Results were presented as mean ± SE; *n* = 3; *p < 0.05; **p < 0.01.

**Fig. 5:**
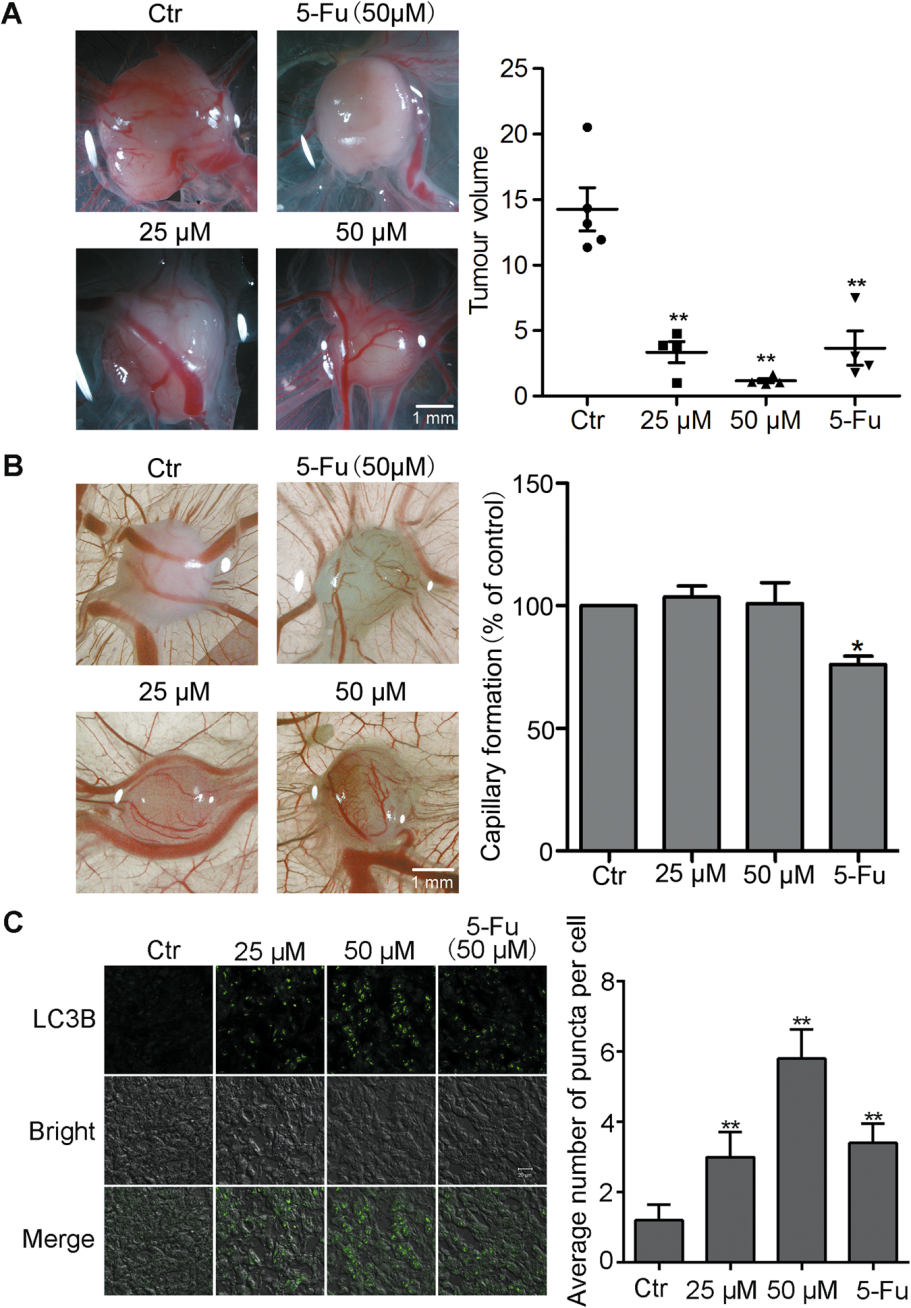
Compound **5e** inhibited the growth of tumor in vivo. **a** Biomicroscopy imaging of control and treated tumors. Bar = 1 mm. **b** Biomicroscopy and quantification of capillary formation with and without compound **5e** and 5-Fu treatment on gelatin sponge. Bar = 1 mm. **c** Immunofluorescence of LC3B-II in the frozen sections of day 6 experimental tumors. Bar = 20 μm. Results were presented as mean ± SE; *n* = 3; *p < 0.05, **p < 0.01.

3-Benzyl-5-((2-nitrophenoxy)methyl)-dihydrofuran-2(3H)-one (3BDO), which was found by our group, could activate mTOR by targeting FKBP12 (FK506-binding protein 1A)^46^. To understand how **5e** inhibited mTOR, we examined the effect of **5e** on RPS6KB1 and 4EBP1 phosphorylation in the presence or absence of 3BDO. As expected, levels of p-RPS6KB1 and p-EIF4EBP1 were decreased with **5e**; however, **5e** failed to decrease the phosphorylation of RPS6KB1 and EIF4EBP1 in the presence of 3BDO (Fig. 4A). Accordingly, the mTOR inhibition of **5e** was reversed by 3BDO. Then, SiFKBP12 was used to investigate the molecule target of **5e**. The interference efficiency of SiFKBP12 was 56% in 60 nM (Fig. S8). As shown in Figs. 4b and 5e could not inhibit the activity of mTOR when FKBP12 was knockdown. These results demonstrated that **5e** inhibited the activity of mTOR via FKBP12.

### Compound **5e** inhibited tumor growth in vivo

Given compound **5e** effectively inhibited the growth of A549 cell and did not influence the growth of HUVECs in vitro (Fig. 1c), we chose the chick embryo CAM model for further research to evaluate the antitumor effect of **5e**. The chick embryo CAM is extensively used for tumor engraftment to evaluate the efficacy of anticancer drugs due to its immune-deficient environment^47^. As shown in Fig. 5a, compared with the DMSO-treated eggs, significant xenograft tumor remission was observed after 6 days in eggs treated with compound **5e**. Notably, compound **5e** exerted a better antitumor effect than the therapeutic drug 5-Fu. We further detected that compound **5e** had no significant toxicity on angiogenesis in vivo in contrast with 5-Fu which suppressed capillary formation (Fig. 5b). Therefore, **5e** effectively inhibited tumor growth in vivo without adverse effect on normal CAM angiogenesis.

### Compound **5e** induced A549 cells autophagy in vivo

To further investigate the mechanism by which compound **5e** inhibited tumor growth in vivo, we prepared frozen sections of solid tumors formed on CAM. Immunofluorescence experiment was performed on frozen sections of tumor. Data showed that compound **5e** elevated the level of LC3B-II in tumor tissue, which was in accordance with the results in vitro (Fig. 5c). These data demonstrated that **5e** inhibited lung cancer growth through inducing autophagy in vivo.

## Discussion

Due to the painful side effects of chemotherapeutic drugs, it is necessary for researchers to improve the selectivity of drugs. Understanding the distribution and target of drugs could be very helpful in improving selectivity. In this study, a series of novel fluorescent compounds were synthesized though sample synthesis steps with high yield. The antiproliferative activity of these compounds against was examined. The results showed that they all showed excellent ability compared with 5-FU. Further SAR analysis showed that the antiproliferative activity of these compounds were mainly affected by aryl group in 5 position of pyrazoline moiety. Further mechanism studied showed that these compounds could arrest cell cycle at the G1 phase and had no influence on cell necrosis.

Using its own fluorescence characteristics, our study found that compound **5e** could selectively accumulate in lysosome. Lysosome decomposition is a very important step of autophagy^48^. Autophagy is an indispensable cellular process for protein and organelle quality control. Autophagy is a double-edged sword. Moderate autophagy could promote cell survival, while excessive autophagy could induce cell death^28,49^. Previous studies have indicated that autophagy suppression may be a therapeutic strategy for cancer treatment^29,50,51^. We found that these compounds could induce autophagy and complete autophagy flux.

**Fig. 6:**
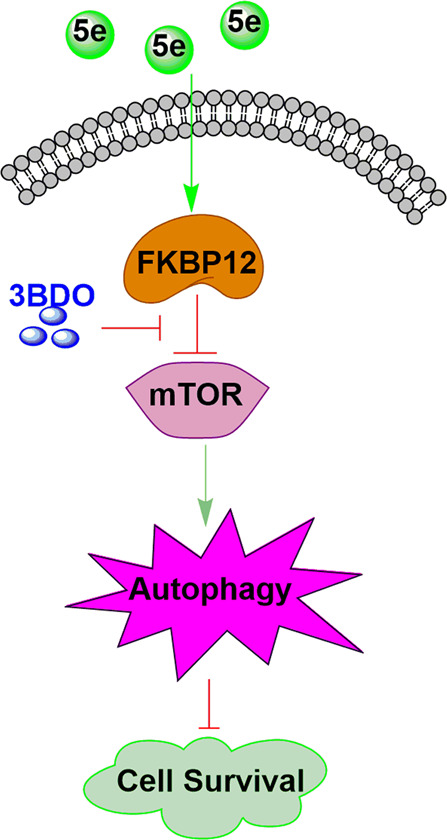
The mechanism of compound 5e in inhibiting cancer cell survival. In A549 cells, compound **5e** can inhibit the activity of mTOR via FKBP12, which could be reversed by **3BDO**. Then autophagy was induced and cellsurvival was inhibited.

mTOR has an essential role in different tissues and cells. As mTOR-mediated signaling associated with many diseases, pharmacological agents that modulate mTOR signaling could be helpful in improving many diseases^52^. mTOR also plays an important role in autophagy^53^. In this study, phosphorylation of RPS6KB1 and EIF4EBP1 was significantly decreased by **5e**, which could be reversed by 3BDO, an mTOR activator, and autophagy inhibitor. **5e** failed to decrease the phosphorylation of RPS6KB1 and EIF4EBP1 when FKBP12 was knockdown. Accordingly, we demonstrated that **5e** inhibited mTOR though targeting FKBP12 (Fig. 6). FKBP12 targets mTOR in complex with rapamycin^54^. 3BDO could dock on FKBP12 in the same sites (TYR82A and ILE56A) as rapamycin^46^. The structure of **5e** is different with 3BDO, so we deduced that **5e** might occupy rapamycin binding sites more easily than 3BDO.

CAM model was chosen for further research to evaluate the antitumor effect of **5e** in vivo. Our results showed **5e** significantly inhibited tumor growth compared with 5-FU and showed no adverse influence of angiogenesis. Immunofluorescence experiment demonstrated that **5e** inhibited lung cancer growth through inducing autophagy in vivo.

In conclusion, we found a series of fluorescent thiazole–pyrazoline derivatives, which could inhibit the growth of A549 cells in vitro and inhibit tumor growth in vivo. Compound **5e** induced autophagy though inhibiting the activity of mTOR via FKBP12, which could be reversed by 3BDO, an mTOR activator and autophagy inhibitor. Moreover, compound **5e** showed no adverse influence of normal cells and angiogenesis. Therefore, our research provides potential lead compounds for the development of new anticancer drugs against human lung cancer.

## Supplementary information

Supplementary Information

Supplementary Information 2

Supplementary Information 3

Supplementary Information 4

Supplementary Information 5

Supplementary Information 6

Supplementary Information 7

Supplementary Information 8

Supplementary Information 9

Supplementary Information 10

Supplementary Information 11
