# Supplementary material for: Discovery of new fluorescent thiazole–pyrazoline derivatives as autophagy inducers by inhibiting mTOR activity in A549 human lung cancer cells
Source: Cell Death Dis. 2020 Jul 20;11(7):551. doi: 10.1038/s41419-020-02746-w (PMC7371735; doi:10.1038/s41419-020-02746-w)
Supplement: Supplementary file 1 — Supplementary Information [file 41419_2020_2746_MOESM1_ESM.docx]

Supplementary information

**Discovery of new** **fluorescent thiazole-pyrazoline derivatives as autophagy inducers by inhibiting mTOR activity in A549 human lung cancer cells**

ZhaoMin Lin^1^, ZhaoYang Wang^2^, XueWen Zhou^3^, Ming Zhang^1^, DongFang Gao^1^, Lu Zhang^1^, Peng Wang^1^, Yuan Chen^1^, YuXing Lin^1^, BaoXiang Zhao^3^, JunYing Miao^2^, Feng Kong^4,*^

*^1^ Institute of Medical Science, the Second Hospital of Shandong University, Jinan 250033, P.R. China.*

*^2^ Shandong Provincial Key Laboratory of Animal Cells and Developmental Biology, School of Life Science, Shandong University, Jinan 250100, P.R. China*

*^3^ Institute of Organic Chemistry, School of Chemistry and Chemical Engineering, Shandong University, Jinan 250100, P.R. China*

*^4^* *Department of Central Laboratory, Shandong Provincial Hospital affiliated to Shandong University, Jinan 250021, P.R. China.*

*Correspondence to: Dr. Feng Kong, Department of Central Laboratory, Shandong Provincial Hospital affiliated to Shandong University, Jinan 250021, P.R. China.

Fax: + 86 531 68778285; Tel.: + 86 531 68778285.

E-mail address: kongfeng@sdu.edu.cn.

**Spectroscopy data of compounds 5a-5j**

2-(3,5-Diphenyl-4,5-dihydro-1H-pyrazol-1-yl)-4-(pyridin-4-yl)thiazole (**5a**)

Light yellow crystals, 66.9% yield (256 mg). mp: 211–212 °C. ^1^H-NMR (DMSO-*d6*, 300 MHz), 8.54 (dd, *J* = 4.7, 1.4 Hz, 2H), 7.83–7.80 (m, 2H), 7.71 (s, 1H), 7.59 (dd, *J* = 4.5, 1.5 Hz, 2H), 7.52–7.26 (m, 8H), 5.70 (dd, *J* = 11.7, 6.3 Hz, 1H), 4.07 (dd, *J* = 18.0, 12.0 Hz, 1H), 3.40 (dd, *J* = 18.0, 6.6 Hz, 1H). ^13^C-NMR (DMSO-*d6*, 75 MHz) 43.70, 64.72, 109.35, 120.18 (2C), 126.95 (2C), 127.14 (2C), 128.07, 129.07(2C), 129.32 (2C), 130.57, 131.33, 141.52, 142.13, 148.54, 150.47(2C), 153.82, 165.14. HRMS (C_23_H_19_N_4_S): Calcd. [M+H]^+^: 383.1330; found value: 383.1323.

2-(3-(4-Chlorophenyl)-5-phenyl-4,5-dihydro-1H-pyrazol-1-yl)-4-(pyridin-4-yl)thiazole (**5b**)

Light yellow crystals, 72.5% yield (300 mg). mp: 217–218 °C. ^1^H-NMR (DMSO-*d6*, 400 MHz), 8.53 (d, *J* = 6.0, 2H), 7.82 (d, *J* = 8.5 Hz, 2H), 7.71 (s, 1H), 7.59 (dd, *J* = 26.5, 7.3 Hz, 4H), 7.48–7.33 (m, 4H), 7.28 (t, *J* = 7.1 Hz, 1H), 5.71 (dd, *J* = 11.9, 6.6 Hz, 1H), 4.07 (dd, *J* = 18.1, 12.8 Hz, 1H), 3.40 (dd, *J* = 18.0, 6.7 Hz, 1H). ^13^C-NMR (DMSO-*d6*, 75 MHz) 43.55, 64.94, 109.44, 120.17 (2C), 127.18 (2C), 128.11, 128.65 (2C), 129.06 (2C), 129.39 (2C), 130.27, 135.07, 141.46, 141.98, 148.58, 150.50 (2C), 152.79, 165.01. HRMS (C_23_H_18_ClN_4_S): Calcd. [M+H]^+^: 417.0941; found value: 417.0942.

2-(3-(4-Methoxyphenyl)-5-phenyl-4,5-dihydro-1H-pyrazol-1-yl)-4-(pyridin-4-yl)thiazole (**5c**)

Yield 59.2%, yellow solid, mp: 242–243 °C. ^1^H-NMR (400 MHz, DMSO-*d6*) δ 8.53 (dd, *J* = 4.6, 1.4 Hz, 2H), 7.82 (d, *J* = 8.8 Hz, 2H), 7.67 (s, 1H), 7.63 (dd, *J* = 4.6, 1.4 Hz, 4H), 7.43-7.28 (m, 5H), 7.05 (d, *J* = 8.9 Hz, 1H), 5.66 (dd, *J* = 11.8, 6.4 Hz, 1H), 4.05 (dd, *J* = 17.9, 11.8 Hz, 1H), 3.83 (s, 1H), 3.36 (dd, *J* = 17.9, 6.4 Hz, 1H). HRMS calcd for C_24_H_21_N_4_OS [M+H]^+^: 413.1436, found: 413.1430.

2-(5-(4-Methoxyphenyl)-3-phenyl-4,5-dihydro-1H-pyrazol-1-yl)-4-(pyridin-4-yl)thiazole (**5d**)

Yield 72.3%, yellow solid, mp: 178–179 °C. ^1^H-NMR (400 MHz, DMSO-*d6*) δ 8.55 (d, *J* = 6.0 Hz, 2H), 7.82-7.48 (m, 8H), 7.36 (d, *J* = 8.7 Hz, 2H), 6.93 (d, *J* = 8.7 Hz, 2H), 5.65 (dd, *J* = 11.8, 6.4 Hz, 1H), 4.05 (dd, *J* = 17.9, 11.8 Hz, 1H), 3.72 (s, 1H), 3.36 (dd, *J* = 17.9, 6.4 Hz, 1H). HRMS calcd for C_24_H_21_N_4_OS [M+H]^+^: 413.1436, found: 413.1434.

2-(3-(4-Chlorophenyl)-5-(4-methoxyphenyl)-4,5-dihydro-1H-pyrazol-1-yl)-4-(pyridin-4-yl)thiazole (**5e**)

Yield 71.7%, yellow solid, mp: 193–194 °C. ^1^H-NMR (400 MHz, DMSO-*d6*) δ 8.55 (dd, *J* = 4.6, 1.4 Hz, 2H), 7.81 (d, *J* = 8.6 Hz, 2H), 7.70 (s, 1H), 7.66 (dd, *J* = 4.6, 1.4 Hz, 2H), 7.56 (d, *J* = 8.6 Hz, 2H), 6.93 (d, *J* = 8.7 Hz, 2H), 5.66 (dd, *J* = 12.0, 6.4 Hz, 1H), 4.03 (dd, *J* = 18.0, 12.0 Hz, 1H), 3.72 (s, 1H), 3.36 (dd, *J* = 18.0, 6.4 Hz, 1H). HRMS calcd for C_24_H_20_ClN_4_OS [M+H]^+^: 447.1046, found: 447.1043.

2-(3,5-Bis(4-methoxyphenyl)-4,5-dihydro-1H-pyrazol-1-yl)-4-(pyridin-4-yl)thiazole (**5f**)

Light yellow crystals, 71.7% yield (320 mg). mp: 200–201 °C. ^1^H-NMR (DMSO-*d6*, 300 MHz), 8.54 (dd, *J* = 4.8, 1.5 Hz, 2H), 7.75(d, *J* = 8.7 Hz, 2H), 7.68–7.66 (m, 3H), 7.34 (d, *J* = 8.7, 2H), 7.05(d, *J* = 8.7 Hz, 2H), 6.92 (d, *J* = 8.7 Hz, 2H), 5.61 (dd, *J* = 11.7, 6.2 Hz,1H), 4.01 (dd, *J* = 17.9, 11.8 Hz, 1H), 3.82 (s, 3H), 3.72 (s, 3H), 3.38 (dd, *J* = 17.9, 6.3 Hz, 1H). ^13^C-NMR (DMSO-*d6*, 100 MHz) 43.70, 55.51, 55.82, 64.00, 108.94, 114.35 (2C), 114.76 (2C), 120.20 (2C), 123.92, 128.49 (2C), 128.60 (2C), 134.11, 141.58, 148.55, 150.51(2C), 153.63, 159.10, 161.26, 165.21. HRMS (C_25_H_23_N_4_O_2_S): Calcd. [M+H]^+^: 443.1542; found: 443.1540.

2-(5-(Benzo[d][1,3]dioxol-5-yl)-3-phenyl-4,5-dihydro-1H-pyrazol-1-yl)-4-(pyridin-4-yl)thiazole (**5g**)

Yield 71.9%, yellow solid, mp: 183–184 °C. ^1^H-NMR (400 MHz, DMSO-*d6*) δ 8.55 (dd, *J* = 4.7, 1.4 Hz, 2H), 7.81-7.79 (m, 2H), 7.71 (s, 1H), 7.67 (dd, *J* = 4.7, 1.5 Hz, 2H), 7.52-7.48 (m, 3H), 6.95-6.89 (m, 3H), 5.98 (d, *J* = 5.8 Hz, 2H), 5.63 (dd, *J* = 11.8, 6.5 Hz, 1H), 4.01 (dd, *J* = 18.0, 11.8 Hz, 1H), 3.38 (dd, *J* = 18.0, 6.5 Hz, 1H). HRMS calcd for C_24_H_19_N_4_O_2_S [M+H]^+^: 427.1229, found: 427.1221.

2-(5-(Benzo[d][1,3]dioxol-5-yl)-3-(4-chlorophenyl)-4,5-dihydro-1H-pyrazol-1-yl)-4-(pyridin-4-yl)thiazole (**5h**)

Yield 45.1%, yellow solid, mp: 204–205 °C. ^1^H-NMR (400 MHz, DMSO-*d6*) δ 8.55 (d, *J* = 5.9 Hz, 2H), 7.80 (d, *J* = 8.5 Hz, 2H), 7.71 (s, 1H), 7.67 (d, *J* = 5.9 Hz, 2H), 7.56 (d, = 8.5 Hz, 2H), 6.95-6.89 (m, 3H), 5.98 (d, *J* = 6.7 Hz, 2H), 5.64 (dd, *J* = 11.8, 6.5 Hz, 1H), 4.01 (dd, *J* = 18.0, 11.8 Hz, 1H), 3.38 (dd, *J* = 18.0, 6.5 Hz, 1H). HRMS calcd for C_24_H_18_ClN_4_O_2_S [M+H]^+^: 461.0839, found: 461.0833.

2-(5-(Benzo[d][1,3]dioxol-5-yl)-3-(4-methoxyphenyl)-4,5-dihydro-1H-pyrazol-1-yl)-4-(pyridin-4-yl)thiazole (**5i**)

Yield 44.6%, yellow solid, mp: 188–189 °C. ^1^H-NMR (400 MHz, DMSO-*d6*) δ 8.55 (d, *J* = 5.9 Hz, 2H), 7.74 (d, *J* = 8.5 Hz, 2H), 7.67-7.66 (m, 3H), 7.05 (d, *J* = 8.8 Hz, 2H), 6.93-6.88 (m, 3H), 5.98 (d, *J* = 6.0 Hz, 2H), 5.59 (dd, *J* = 11.6, 6.4 Hz, 1H), 3.99 (dd, *J* = 17.8, 11.6 Hz, 1H), 3.82 (s, 3H), 3.34 (dd, *J* = 17.8, 6.4 Hz, 1H). HRMS calcd for C_25_H_21_N_4_O_3_S [M+H]^+^: 457.1334, found: 457.1328.
