# Supplementary material for: Discovery of new fluorescent thiazole–pyrazoline derivatives as autophagy inducers by inhibiting mTOR activity in A549 human lung cancer cells
Source: Cell Death Dis. 2020 Jul 20;11(7):551. doi: 10.1038/s41419-020-02746-w (PMC7371735; doi:10.1038/s41419-020-02746-w)
Supplement: Supplementary file 2 — Supplementary Information 2 [file 41419_2020_2746_MOESM2_ESM.docx]

**Figure S1. The morphological change of A549 cells**

Cells were treated with compounds **5a**-**5i** at the indicated concentrations for 12, 24 and 48 h. 0.1% DMSO was used as control (100×). Bar = 20 μm.

**Figure S2. Effects of compounds 5a-5i on Cell Cycle arrest and LDH leakage.**

(**A**) A549 cells were treated with compounds **5a**, **5d**, **5e**, **5g** and **5h** at the concentration of 10 μM for 48 h, then, the cell cycle distribution was analyzed by flow cytometry. (**B**) Cells were treated with the compounds for 48 h at the concentration of 10 μM, and then the culture medium was collected for LDH assay. Results were presented as mean ± SE; n = 3; **p* <0.05.

**Figure S3. The fluorescence characteristics of compounds 5a-5i**

A549 cells were treated with compounds **5a-5i** at indicated concentrations for 24 h and photographed under different excitation light and bright light (100×). Bar = 10 μm.

**Figure S4. Compounds 5a-5i induced autophagy**

(**A**) Western blot analysis of LC3B-II/I in A549 cells treated with **5a**-**5i** at 10 μM for 12 h and quantification of LC3B-II level. (**B**) Western blot analysis of LC3B-I and LC3B-II in A549 cells treated with **5a**-**5i** at 10 μM for 24 h and quantification of LC3B-II level. β-actin was used as a loading control. (**C**) Images of EGFP-LC3B dots in U87 cells. U87 cells were treated with compounds **5a**-**5i** at the concentration of 10 μM or Rapa (4 μM) for 24 h (200×). Rapa group was used as a positive control. Bar = 10 μm. Results were presented as mean ± SE; n = 3; **p* <0.05; ***p* <0.01.

**Figure S5. Inhibition of autophagy rescues cell death in 5e-treated A549 cells.**

The cell death of PC3 cells exposed to **5e** alone or combined treatment (A) PC3 cells treated with 10 μM **5e** or 10 μM **5e** plus 50 nM Bafilomycin-A1 (Baf A1) for 24 h. (B) PC3 cells treated with 10 μM **5e** or 10 μM **5e** plus 20 mM chloroquine (CQ) for 24 h. (C) Knockdown of LC3B by siRNA for 48 h prior to 10 μM **5e** treatment. (D) Western blot analysis of LC3B-II/I in A549 cells after treatment. Results were presented as mean ± SE; n = 3; ***p* <0.01.

**Figure S6.** Western blot analysis of LC3B-II/I in HUVECs after treatment with 10 μM **5e** for indicated time. Results were presented as mean ± SE; n=3.

**Figure S7.** **(A)** Immunofluorescence of p-mTOR and compound **5e** in A549 cells. **(B)** Immunofluorescence of mTOR and compound **5e** in A549 cells. Microscopic photographs (200×) were taken under a confocal laser scanning microscope. Bar = 20 μm.

**Figure S8. The interference efficiency of SiFKBP12**

Western blot analysis of FKBP12 with SiFKBP12 in 30, 60, 90 nM for 24 h. β-actin was used as a loading control. Results were presented as mean ± SE; n=3; **p* <0.05; ***p* <0.01.
