# Supplementary material for: Discovery of new fluorescent thiazole–pyrazoline derivatives as autophagy inducers by inhibiting mTOR activity in A549 human lung cancer cells
Source: Cell Death Dis. 2020 Jul 20;11(7):551. doi: 10.1038/s41419-020-02746-w (PMC7371735; doi:10.1038/s41419-020-02746-w)
Supplement: Supplementary file 3 — Supplementary Information 3 [file 41419_2020_2746_MOESM3_ESM.docx]

**Table S1. Growth inhibitory properties (IC_50_, 48 h) of compound 5e and 5-FU in different cell lines** *^a^*.

| **Compounds** | H460 | HepG-2 | PC3 | 786-O | 4T1 | J82 | HK-2 | 293T |
| --- | --- | --- | --- | --- | --- | --- | --- | --- |
| **5e** | 5.4±0.33 | 3.4±0.26 | 2.5±0.11 | 9.6±0.23 | 4.4±0.25 | 5.1±0.26 | NT *^b^* | NT |
| **5-FU** *^c^* | 18.4±0.12 | 9.4±0.23 | 11.9±0.49 | 15.4±0.22 | 10.8±0.13 | 19.4±0.53 | 25.6±0.66 | 29.8±0.73 |

*^a^* Results are expressed as the mean values of IC_50_ ± SEM in μM. *^b^* “NT” means Not active (IC_50_ >100 μM). *^c^* 5-FU was used as the positive control. H460, human non-small-cell lung cancer cell line; HepG-2, human liver carcinoma cell line; PC3, human hormone-independent prostate carcinoma cell line; 786-O, human kidney clear cell adenocarcinoma cell line; 4T1, human breast carcinoma cell line; J82, human bladder cancer cell line; HK-2, human renal tubular epithelial cell; 293T, human embryonic kidney cell. The experiments were performed three times.
