# Supplementary figures and images for: Discovery of new fluorescent thiazole–pyrazoline derivatives as autophagy inducers by inhibiting mTOR activity in A549 human lung cancer cells
Source: Cell Death Dis. 2020 Jul 20;11(7):551. doi: 10.1038/s41419-020-02746-w (PMC7371735; doi:10.1038/s41419-020-02746-w)

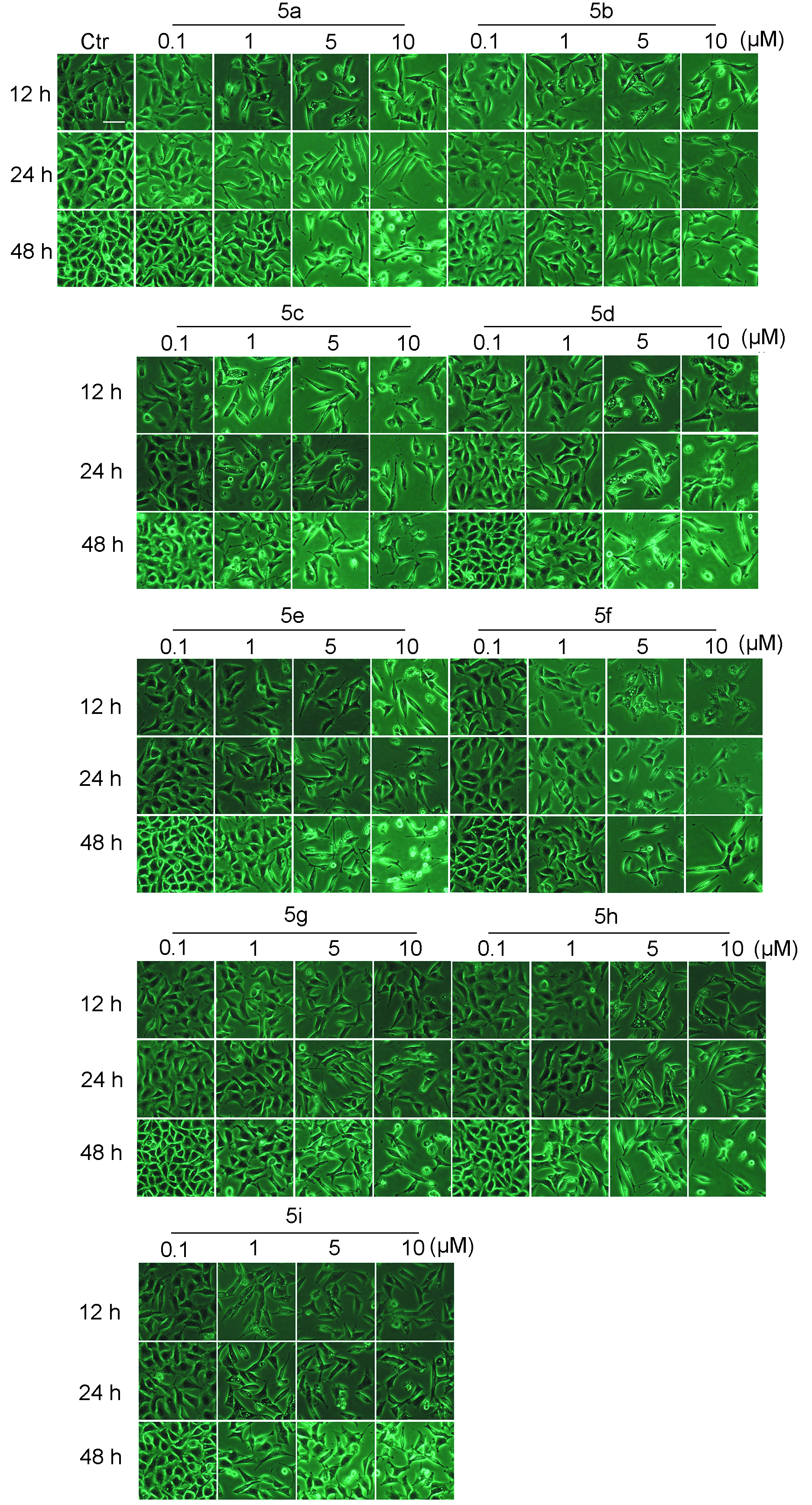

Supplement: Supplementary file 4 — Supplementary Information 4 [file 41419_2020_2746_MOESM4_ESM.tif]

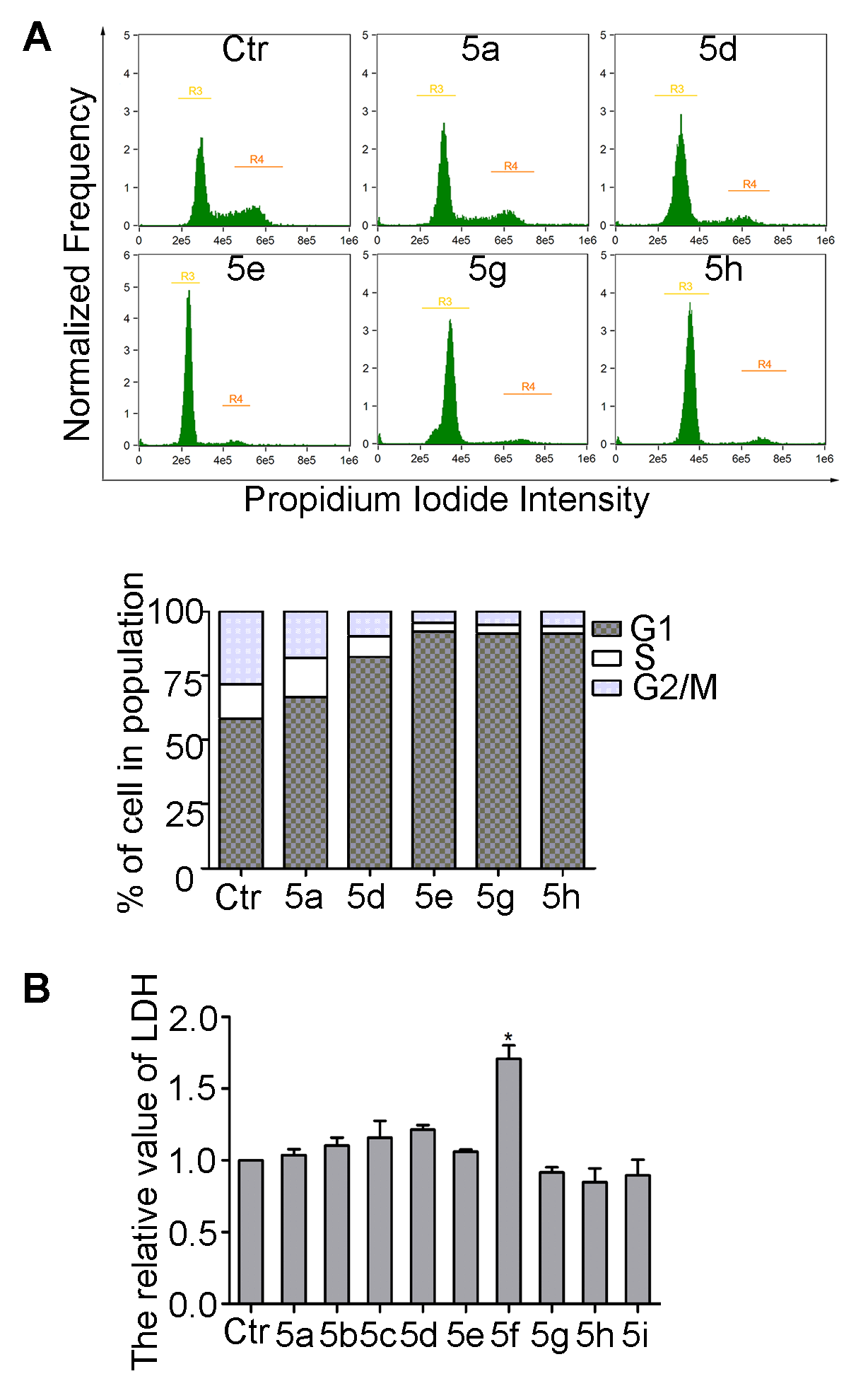

Supplement: Supplementary file 5 — Supplementary Information 5 [file 41419_2020_2746_MOESM5_ESM.tif]

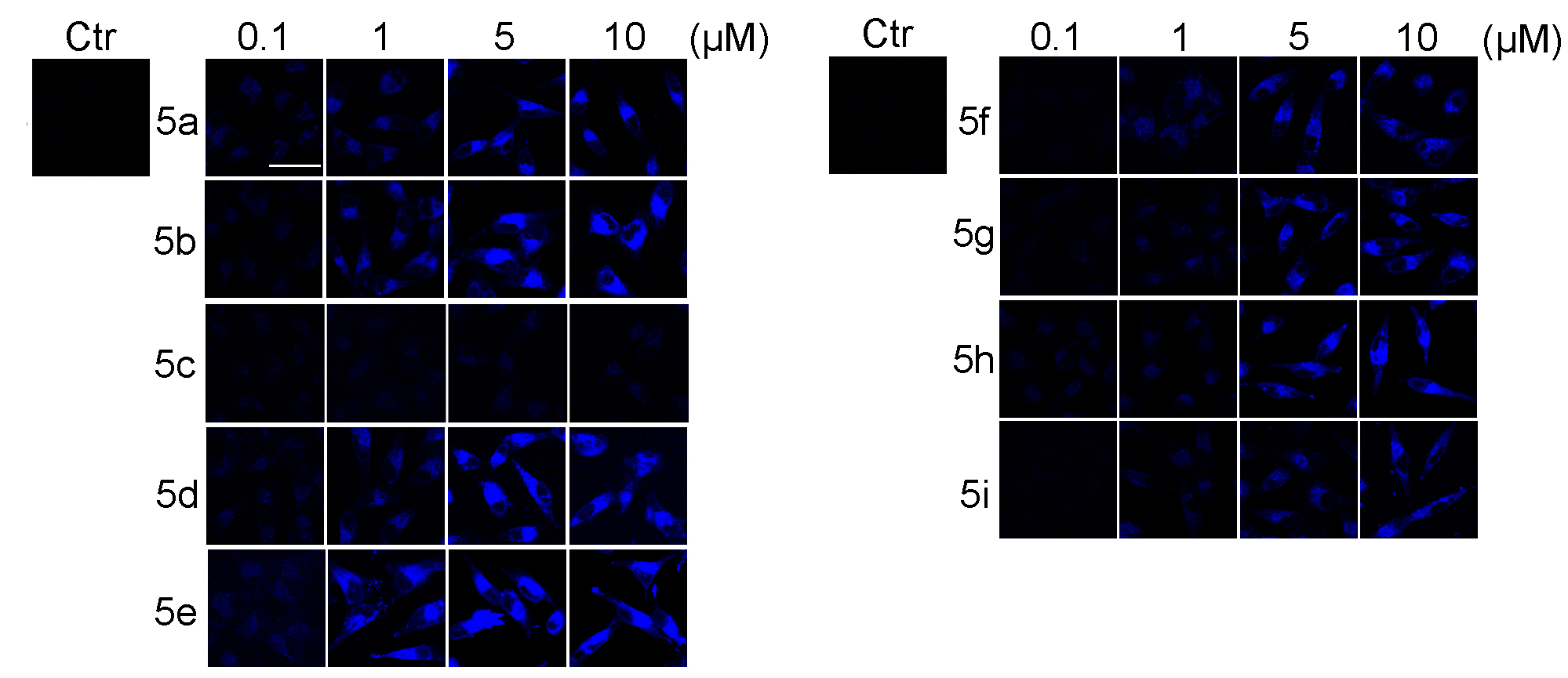

Supplement: Supplementary file 6 — Supplementary Information 6 [file 41419_2020_2746_MOESM6_ESM.tif]

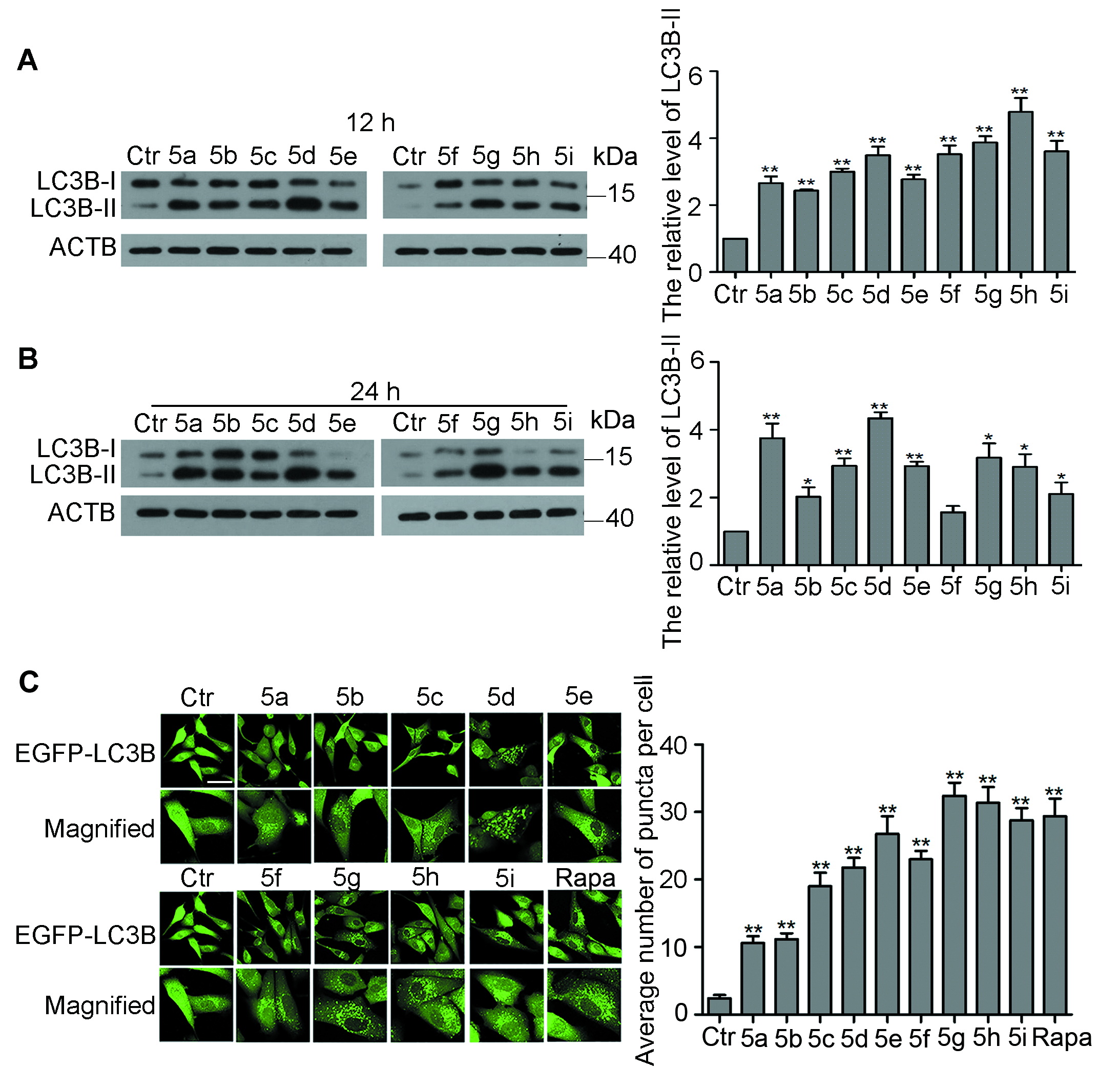

Supplement: Supplementary file 7 — Supplementary Information 7 [file 41419_2020_2746_MOESM7_ESM.tif]

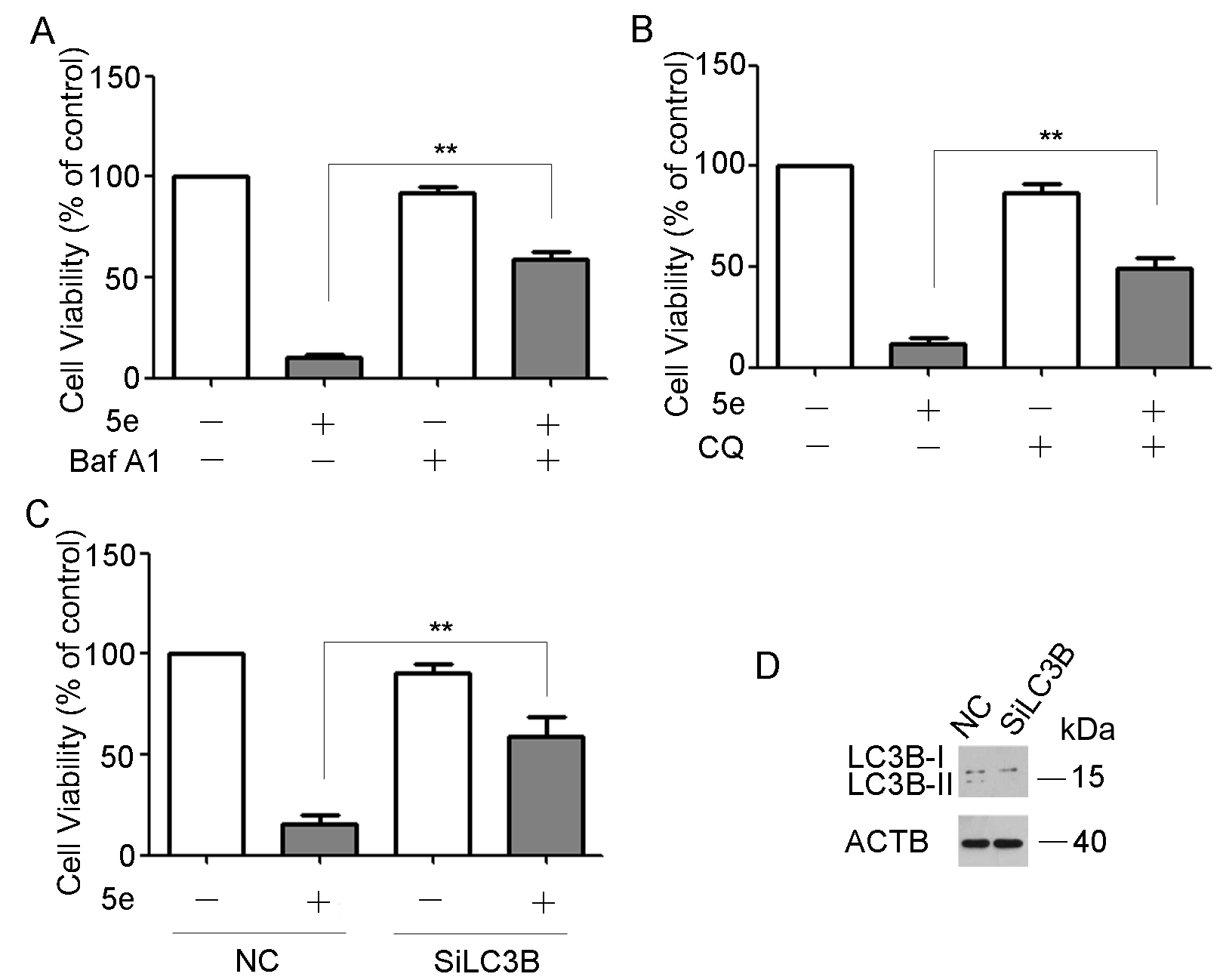

Supplement: Supplementary file 8 — Supplementary Information 8 [file 41419_2020_2746_MOESM8_ESM.tif]

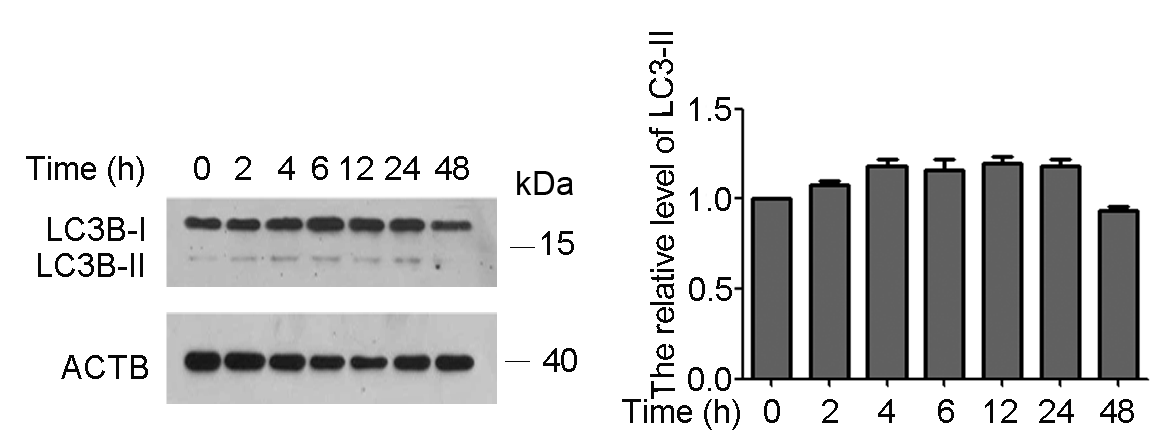

Supplement: Supplementary file 9 — Supplementary Information 9 [file 41419_2020_2746_MOESM9_ESM.tif]

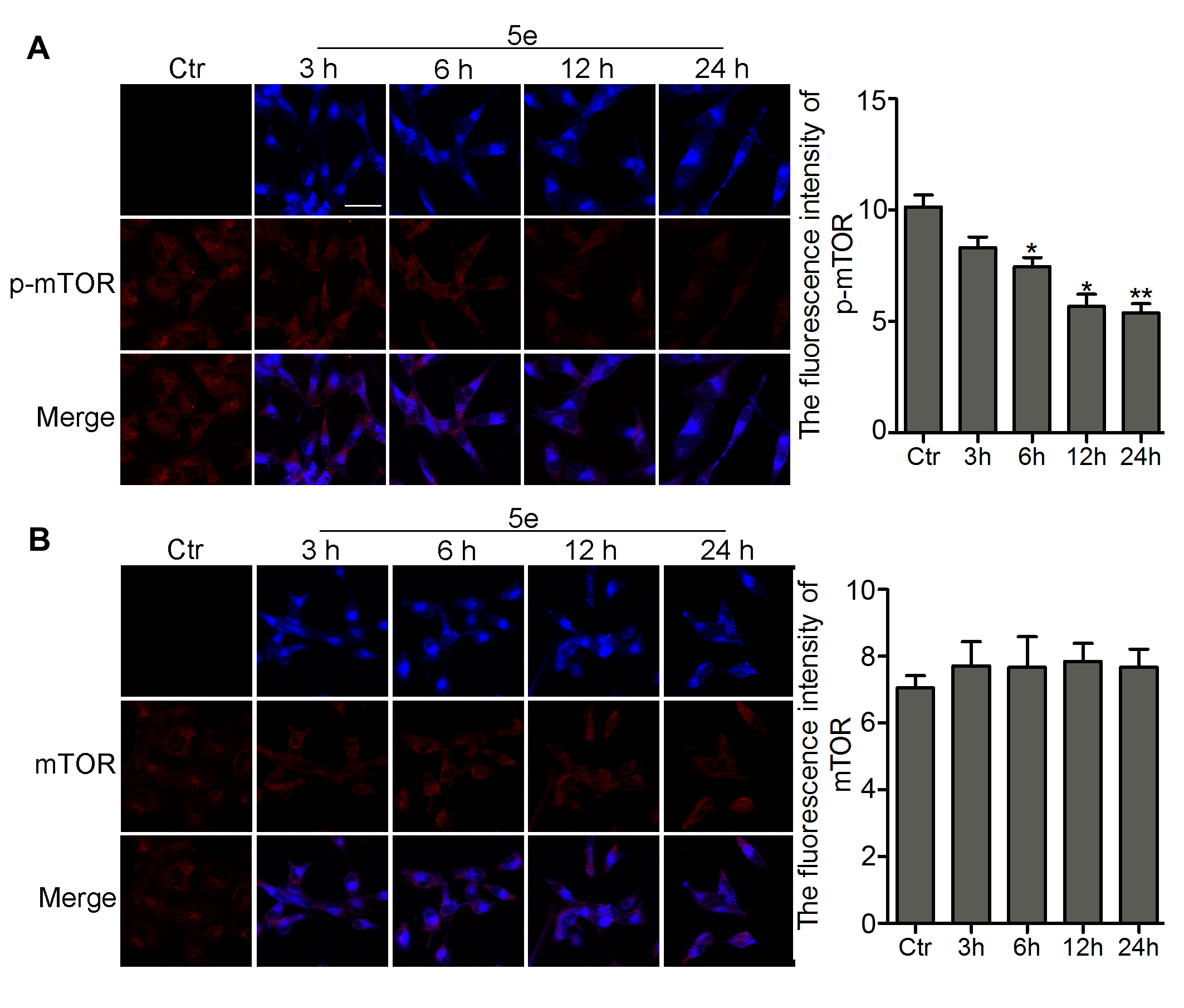

Supplement: Supplementary file 10 — Supplementary Information 10 [file 41419_2020_2746_MOESM10_ESM.tif]

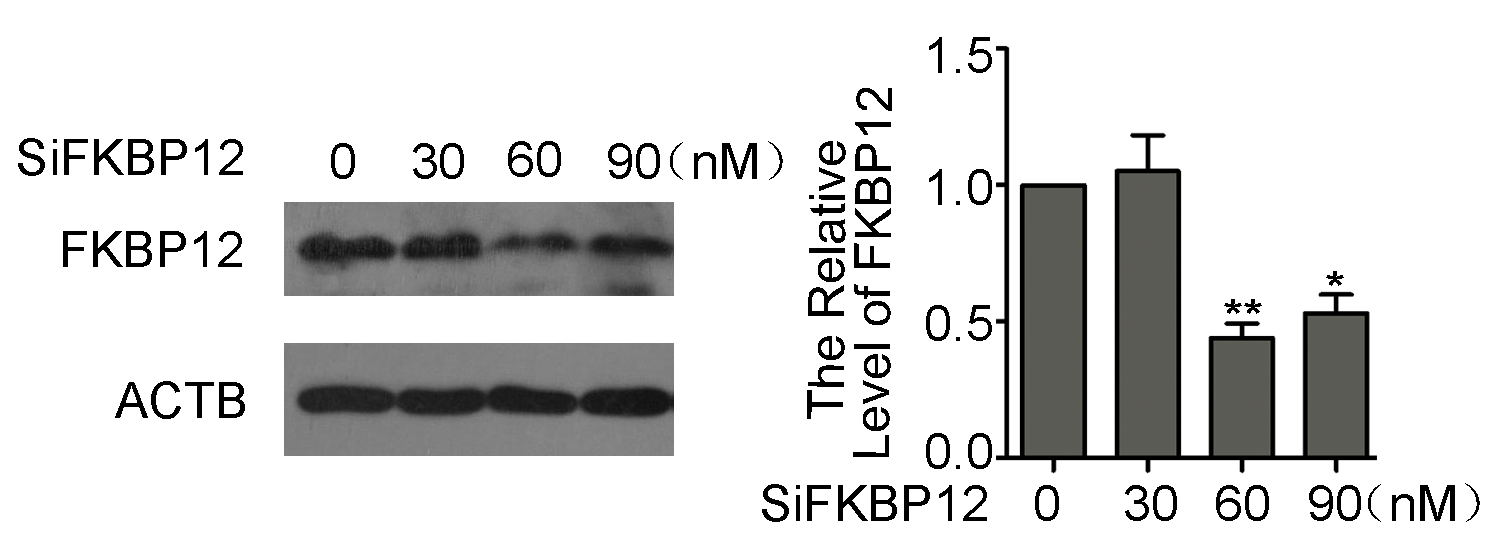

Supplement: Supplementary file 11 — Supplementary Information 11 [file 41419_2020_2746_MOESM11_ESM.tif]
